# Translation Approach for Dentine Regeneration Using GSK-3 Antagonists

**DOI:** 10.1177/0022034520908593

**Published:** 2020-03-10

**Authors:** L.K. Zaugg, A. Banu, A.R. Walther, D. Chandrasekaran, R.C. Babb, C. Salzlechner, M.A.B. Hedegaard, E. Gentleman, P.T. Sharpe

**Affiliations:** 1 Centre for Craniofacial and Regenerative Biology, King’s College London, London, UK; 2 Department of Reconstructive Dentistry, University Center for Dental Medicine, University of Basel, Basel, Switzerland; 3 Department of Chemical Engineering, Biotechnology and Environmental Technology, University of Southern Denmark, Odense, Denmark

**Keywords:** repair, stem cells, mineralization, Raman microspectroscopy, pulp, Wnt signaling

## Abstract

The canonical Wnt/β-catenin signaling pathway is crucial for reparative dentinogenesis following tooth damage, and the modulation of this pathway affects the rate and extent of reparative dentine formation in damaged mice molars by triggering the natural process of dentinogenesis. Pharmacological stimulation of Wnt/β-catenin signaling activity by small-molecule GSK-3 inhibitor drugs following pulp exposure in mouse molars results in reparative dentinogenesis. The creation of similar but larger lesions in rat molars shows that the adenosine triphosphate (ATP)–competitive GSK-3 inhibitor, CHIR99021 (CHIR), and the ATP noncompetitive inhibitor, Tideglusib (TG), can equally enhance reparative dentine formation to fully repair an area of dentine damage up to 10 times larger, mimicking the size of small lesions in humans. To assess the chemical composition of this newly formed dentine and to compare its structure with surrounding native dentine and alveolar bone, Raman microspectroscopy analysis is used. We show that the newly formed dentine comprises equal carbonate to phosphate ratios and mineral to matrix ratios to that of native dentine, both being significantly different from bone. For an effective dentine repair, the activity of the drugs needs to be restricted to the region of damage. To investigate the range of drug-induced Wnt-activity within the dental pulp, RNA of short-term induced (24-h) molars is extracted from separated roots and crowns, and quantitative *Axin2* expression is assayed. We show that the activation of Wnt/β-catenin signaling is highly restricted to pulp cells in the immediate location of the damage in the coronal pulp tissue with no drug action detected in the root pulp. These results provide further evidence that this simple method of enhancement of natural reparative dentinogenesis has the potential to be translated into a clinical direct capping approach.

## Introduction

The formation of reparative dentine bridges in response to tooth damage with pulp exposure is a natural phenomenon serving to protect and repair the tooth (Cvek 1978; Ricucci et al. 2019). The new odontoblast-like cells that produce reparative dentine are formed from resident pulp stem cells mobilized by signals released at the site of damage (Babb et al. 2017). Wnt/β-catenin signaling is required for stem cell mobilization, and genetic titration of its activity modulates the extent of reparative dentine formation (Babb et al. 2017; Neves et al. 2017). Glycogen synthase kinase 3 (GSK-3) is a core intracellular component of the Wnt/β-catenin signaling pathway that phosphorylates Axin and β-catenin (Liu et al. 2002; Zeng et al. 2005; Clevers and Nusse 2012). A range of small-molecule antagonists of GSK-3 have been developed as drugs to activate Wnt-activity in responsive cells (Coghlan et al. 2000; Sato et al. 2004; Clevers and Nusse 2012; Leone et al. 2012). Delivery of GSK-3 inhibitor drugs directly into experimentally created deep cavities in mice on biodegradable collagen sponges results in upregulation of Wnt-activity in pulp stem cells (Neves et al. 2017). The outcome of induced elevated Wnt-activity is a significant enhancement of reparative dentine formation. As the collagen sponge biodegrades, it is replaced by reparative dentine, thus allowing the entire cavity to be filled by reparative dentine (Neves et al. 2017).

In order for these approaches to be translatable into a clinical product that can replace conventional direct pulp capping agents such as mineral trioxide aggregate (MTA), a number of questions need to be addressed, such as the following: what is the volume of reparative dentine that can be produced? What is the range of Wnt-activity induction in the pulp? Is the mineral composition of the reparative dentine sufficiently similar to normal dentine?

We describe here a series of experiments in rodents that set out to address these questions with the overall aim to provide a basis on which to formulate the therapeutic use of locally delivered small-molecule GSK-3 inhibitors to advance dentine regeneration in future clinical research approaches.

## Materials and Methods

### Study Design

The objective of the present study was to translate the established molar damage model from mice (Neves et al. 2017; Babb et al. 2019) to rats to investigate and compare the reparative capability and cellular response of rat pulp cells to GSK-3 inhibitors in a scaled-up damage model. To assess the quality and composition of the newly formed reparative dentine, mice molars were drilled and analyzed with Raman microspectroscopy.

### Animal Information

Adult male Wistar rats (7 wk old) and CD1 mice (6 wk old) were used for in vivo molar damage procedures. All animals used in this study were handled in accordance with the UK Home Office Regulations project license 170/01-0783 and personal license IE6A6EC69 (DC), and, experimental procedures were approved by the King’s College Ethical Review Process.

### Pulp Exposure and Drug Placement

The tooth damage protocol for mice was applied as published elsewhere (Neves et al. 2017; Babb et al. 2019). For rats, the protocol was modified accordingly (Appendix Fig. 1). Briefly, rats were anaesthetized with a solution made of Hypnorm (fentanyl/fluanisone, 0.4 mL/kg; VetaPharma Ltd.), sterile water, and Hypnovel (1 mL/kg; Midazolam-Roche) in a ratio of 2:7:5 and injected at the rate of 2.8 mL/kg intraperitoneally. Teeth were disinfected with 70% ethanol, and a carbide bur (FG¼, Ø0.25 mm; JETBrand) coupled to a high-speed handpiece (Kavo Super Torque LUX 2 640B) was used to drill the cavity and to expose the pulp of the middle cusp. The exposed pulp was capped subsequently with a collagen sponge (1 mm^3^, Kolspon; Eucare Ltd.) supplemented with 0.1 µL GSK-3 inhibitors or 0.1 µL dimethyl sulfoxide (DMSO) diluted in phosphate-buffered saline (PBS) (0.5% v/v) as a control. For rats, 20 µM CHIR99021 (CHIR) (Sigma) and 1 µM Tideglusib (TG) (Sigma) were used (Appendix Fig. 2). Both drugs were dissolved in DMSO and diluted to their final concentration accounting for equivalent DMSO/PBS ratios of 0.5% v/v. For mice, 6-bromo-indirubin-3′-oxime (BIO) was used at the concentration of 50 nM (0.2% v/v DMSO/PBS) as previously described (Neves et al. 2017). The injury site was sealed with glass ionomer cement (GIC) (KetacCEMradiopaque; 3M ESPE). Postoperatively, the rats were given 4 mL Hartmann’s Solution (Aquapharm; Animalcare) subcutaneously and monitored on heat mats until full recovery. Rats were humanely sacrificed after 24 h and 4 wk and mice after 6 wk. All animals were kept on a soft diet following tooth damage.

### Dissection, Micro–Computed Tomography Imaging, and Histological Processing

Dissection and fixation were performed as published elsewhere (Babb et al. 2019). Subsequently, specimens were scanned using a Scanco µCT50 micro–computed tomography (µCT) scanner. Mouse specimens were immobilized in 6-mm scanning tubes using cotton gauze and scanned to produce 2-µm voxel size volumes (70 kVp, 114 µA, and 0.5-mm aluminum filter). Rat specimens were scanned in 14-mm tubes (90 kVp, 66 µA, and 0.5-mm aluminum filter) to give 5-µm voxel size volumes. The scans were automatically scaled at reconstruction using calibration objects provided by the µCT manufacturer, consisting of 5 rods of hydroxyapatite (HA) at concentrations of 0 to 790 mg HA/cm^3^ and the absorption values expressed in Hounsfield units (HU).

Subsequently, teeth were decalcified at 4°C for 4 wk in 19% EDTA (pH 7.4), washed in PBS, and processed for paraffin sectioning. Wax blocks were sectioned at 8 µm, mounted on glass slides, and stained with Masson’s trichrome. Bright-field images were taken at 10×, 20×, and 40× magnification.

### Micro-CT Analysis: Damage Dimensions, Mineral Content, and Dentine Volume

To compare both animal models, rat (*n* = 4) and mouse (*n* = 6) teeth were analyzed that lost their capping at early stages. Sagittal and coronal damage dimensions and the corresponding damage area of drilled superior first molars were measured on 2-dimensional images (2D) obtained from µCT scans using the Microview software package (Parallax Innovations). The line function was used to measure the maximum distance of sagittal and transversal cross sections, while the advanced region of interest (ROI) spline function was used to assess the damage area. Standardized contrast settings were set to window/level values of 20,500/12,000 (mouse) and 13,000/10,000 (rat).

Mineral content and dentine volume of newly formed reparative dentine was measured from rat teeth of the TG (*n* = 3), CHIR (*n* = 3), and DMSO (*n* = 4) groups. A representative ROI was selected including the newly formed dentine below the damage area. The ROI was set at *x* = 0.60 mm, *y* = 0.45 mm, and *z* = 0.25 mm; auto threshold was applied and bone density (HA) was set at 5,343 HU and water at −1,000 HU. An unpaired 2-tailed *t* test was used to determine statistical differences of mineral content formation, dentine volume, and damage size/area using the software program GraphPad Prism 7 (version 7.0b; GraphPad Software). A *P* value <0.05 was considered statistically significant.

### Pulp Tissue Extraction for Localized Axin2 Gene Expression Analysis (Quantitative Reverse Transcription Polymerase Chain Reaction)

Pulp tissue was extracted of short-term induced (24-h) superior first rat molars for further relative *Axin2* gene expression analysis at the coronal and root level using 20 µM CHIR, 1 µM TG, 0.5% DMSO, and nondrilled teeth as control (*n* = 4 per group).

Extracted teeth were kept in ice-cold PBS and all 5 roots were separated at the crown-root junction and stored separately from the crowns. The pulp chamber floor was lifted with 27-gauge needles and the coronal pulp tissue was gently extracted with 30-gauge needles. Roots were split using a 27-scalpel and the pulp tissue was scraped with fine tweezers. The tissues were immediately collected in microcentrifuge tubes containing 1.4 mm ceramic spheres (Lysing Matix D, FastPrep 24; MP Biomaterials) and stored on dry ice. Samples were transferred to −80°C until RNA extraction. Total RNA was extracted using TRIzol (Thermo Fisher Scientific) as recommended by the manufacturer recommendations and quantified using NanoDrop (Thermo Fisher Scientific).

Reverse transcription of the RNA was performed using random primers (M-MLV Reverse Transcriptase Kit; Promega) as indicated by the manufacturer instructions, and quantitative reverse transcription polymerase chain reaction gene expression was assayed (LightCycler 480 SYBR Green I Master; Roche). Beta-actin was used as housekeeping gene (3′-GGCTG TATTCCCCTCCATCG-5′ and 5′-CCAGTTGGTAACAATG CCTGT-3′) and Axin2 as target gene (3′-TGACTCTCCTTC CAGATCCCA-5′ and 5′-TGCCCACACTAGGCTGACA-3′). Reactions were performed in triplicates, and relative gene expression was calculated using the 2^-ΔΔC^_T_ method where C_T_ is the threshold cycle. An unpaired 2-tailed *t* test was used to assess significant differences.

### Raman Microspectroscopy

Fixed and scanned specimens were stored at 4°C (sterile PBS + 1% Antibiotic / Antimycotic Solution [ABAM, Sigma]) until Raman microspectroscopy. Teeth were trimmed using a diamond bur (830L;3224; Intensive SA) coupled to a high-speed handpiece under a 20× magnification microscope (Leica MZFLIII). Images were taken and matched with a 2D µCT cross section to facilitate orientation during Raman microspectroscopy (Appendix Fig. 4A, B).

A commercialized Senterra Raman microscope (Bruker; 100-mW, 785-nm laser; Peltier cooled 1,024 × 127 pixels iDUS401 CCD; Andor) was used according to the dimensions given in Appendix Figure 4C. Spectra and white light images of the teeth immersed in PBS were collected through a 10×, N.A. 0.3 (UMPlanFLN; Olympus) water immersion objective. In total, 1,200 lines/mm grating was used, giving a dispersion of ~3 cm^–1^ to 5 cm^–1^ per pixel covering the wavenumber range of 440 cm^–1^ to 1,800 cm^–1^. The system was internally precalibrated using a neon lamp and manually checked using the silicon spectral line at 520.7 cm^–1^. Four hundred Raman spectra were collected using a motorized stage in a matrix of 40 × 10 positions on each tooth encompassing both native dentine and defect in an area of ~500 × 300 µm^2^. One hundred spectra of alveolar bone were collected beneath the tooth in a matrix of 10 × 10 positions covering an area of ~200 × 200 µm^2^. Each spectrum was acquired by a 5-s integration time and 4 accumulations.

### Raman Data Processing

All spectral processing was performed using in-house written methods through MATLAB software (2017; MathWorks). Preprocessing involved baseline correction using asymmetric least squares smoothing (Eilers 2003; Peng et al. 2010) and vector normalization to remove instrument effects. Spectra representing glass ionomer cement were removed from the data set before further analysis. Principal component analysis (PCA) was performed on the entire data set of preprocessed Raman spectra from 6 treated teeth and 1 control (untreated). PCA scores were linearly transformed into false colors using minimum-maximum feature scaling. Spectral features were extracted using Gaussian fits to the data (Morris and Mandair 2011). The position, full width half maximum (FWHM), and area of the main hydroxyapatite 960-cm^–1^ (ν_1_PO_4_^3–^) phosphate peak were determined using 2 Gaussian fits. To estimate the collagen matrix content, the area of the 853-cm^–1^ to 875-cm^–1^ (νC-C) band was determined using 2 Gaussian fits. A single Gaussian fit was used for estimating the area of the 1,070-cm^–1^ (ν_1_CO_3_^2–^) band and used as a measure of hydroxyapatite carbonate content (Wallace et al. 2009). Carbonate to phosphate ratios and mineral to matrix ratios were calculated by division of respective band areas. Native dentine, reparative dentine, and bone were compared using Kruskal-Wallis nonparametric analysis of variance (ANOVA) followed by the Wilcoxon rank-sum test for comparison of individual groups (***P *<* 0.001).

## Results

### Reparative Dentine Promotion in Rat Molars

**Figure 1.**
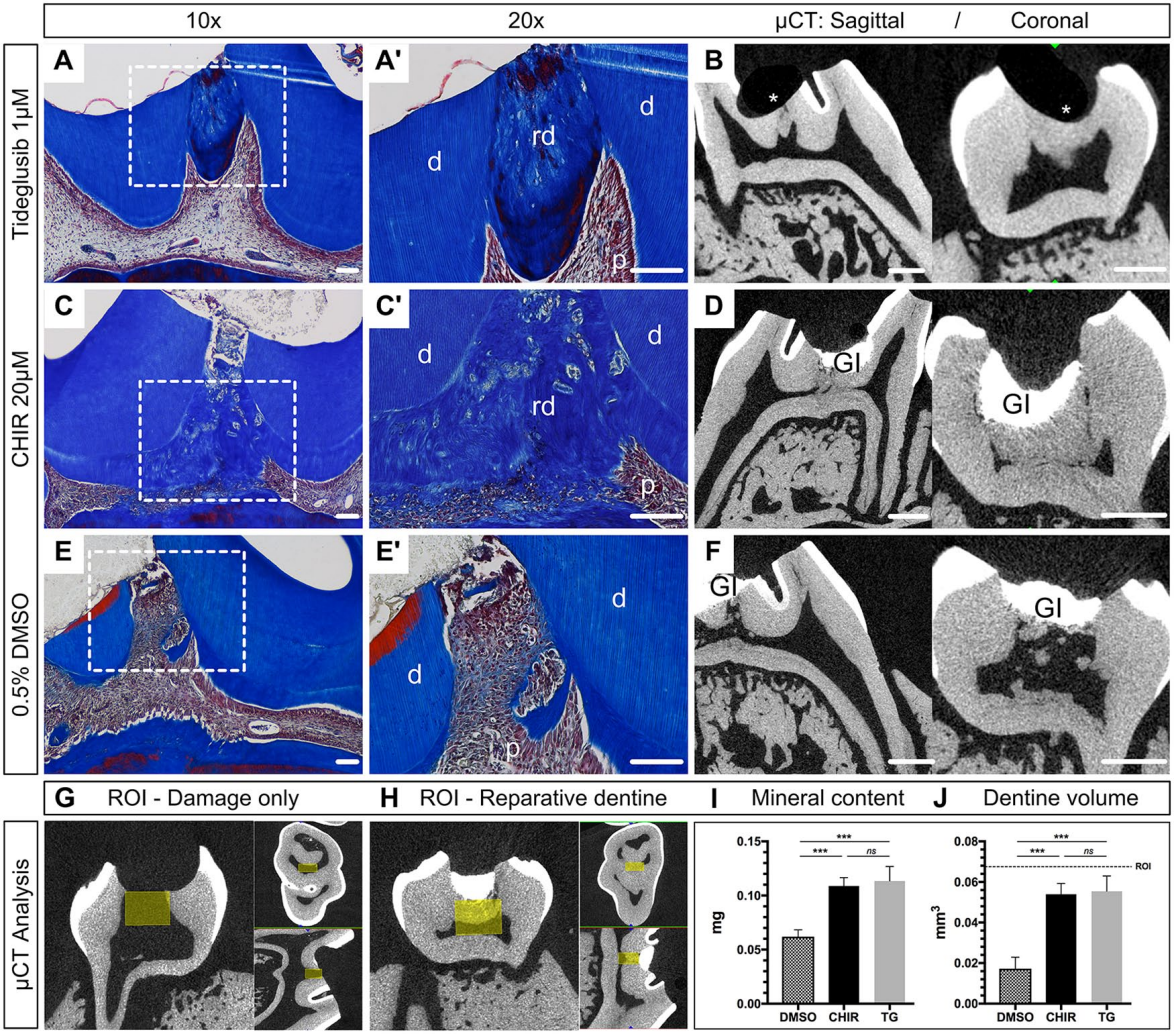
Histology and micro–computed tomography (µCT) analysis of reparative dentinogenesis using GSK-3 inhibitors. (**A–F**) Masson’s trichrome staining and corresponding µCT images of superior first molars 4 wk after pulp damage and placement of collagen sponge containing 0.1 µL of (A, B) Tideglusib (TG) 1 µM (*n* = 3), (C, D) CHIR99021 (CHIR) 20 µM (*n* = 3), or (E, F) dimethyl sulfoxide (DMSO) 0.5% (*n* = 4). (**G–J**) µCT analysis of newly formed dentine (mg) and its corresponding volume (mm^3^) in relation to the selected region of interest (ROI). (G) A µCT scan of a damaged tooth without repair is basis for the definition of a ROI (yellow box: *x* = 0.60 mm; *y* = 0.45 mm; *z* = 0.25 mm) for the subsequent mineral content analysis. (H) The ROI of a representative sample shows that most of the newly formed dentine is included in the selected volume. (I) TG and CHIR present significant higher mineralized tissue with 0.113 mg (±0.013 mg) and 0.109 mg (±0.008 mg) compared to DMSO (0.062 ± 0.006 mg) (*P* < 0.001). (J) No difference is observed in the volume of the newly formed dentine between CHIR and TG, but both are significantly greater than using DMSO (*P* < 0.0003). An unpaired 2-tailed *t* test was used to assess significant differences between groups. Scale bars in A, C, and E are equivalent to 100 µm; scale bars in B, D, and F are equivalent to 500 µm; error bars show SD. d, dentine; GI, glass ionomer filling; p, pulp; rd, reparative dentine; star, missing GI.

Micro-CT images and corresponding histology sections revealed dentine bridge formations in both 1 µM TG and 20 µM CHIR (Fig. 1A–D), whereas no bridge formation occurred in the control group (0.5% DMSO; Fig. 1E, F). In the DMSO group, all samples showed various mineralization loci scattered within the pulp chamber indicating spontaneous mineralization, mainly attributed to parts of detached or transported native dentine (Fig. 1E′). Excessive collagen deposition was present in DMSO, while TG and CHIR exhibited a clear cutoff beneath the repaired damage area exhibiting organized pulp mass and aligned odontoblast-like cells at the dentine-pulp interface. Overall, repaired samples and samples with intact top sealing showed vital pulp tissue with no periapical pathology.

To quantify the newly formed reparative dentine, a ROI was defined (Neves et al. 2017) and scaled up according to the current damage dimensions (Fig. 1G, H). Mineral content analysis revealed highest mineral deposition for TG (0.113 ± 0.013 mg) and CHIR (0.109 ± 0.008 mg) followed by DMSO (0.062 ± 0.006 mg). No significant difference was present between the test drugs, but both TG and CHIR revealed significantly more mineralized tissue compared to the DMSO control group (*P* *<* 0.001) (Fig. 1I). The newly formed dentine volume comprised 0.055 ± 0.007 mm^3^ for TG, 0.054 ± 0.005 mm^3^ for CHIR, and 0.017 ± 0.006 mm^3^ for DMSO, indicating no difference between TG and CHIR but significantly more dentine volume compared to DMSO (*P* < 0.003) (Fig. 1J).

**Figure 2.**
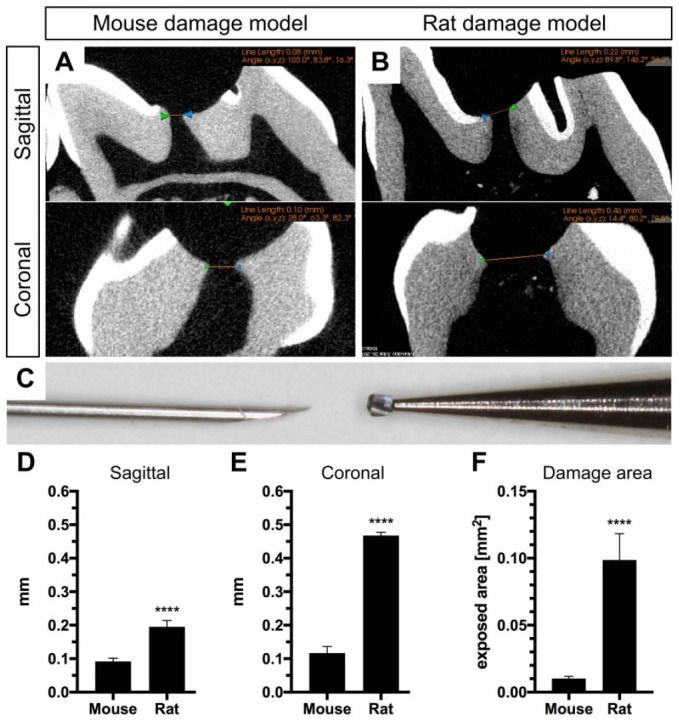
Comparison of animal models. Micro–computed tomography images obtained during the establishment of the molar damage model of both species are used to measure and compare the maximum transversal and sagittal diameter of the exposed pulp of superior first molars in (**A**) mice (*n* = 6) and (**B**) rats (*n* = 4). (**C**) Pulp damage in mice is performed with the tip of a 30-gauge needle (±0.1 mm) and with a 0.25-mm carbide bur in rats. (**D**) The sagittal width analysis reveals that the exposed pulp in rats is smaller than the diameter of the bur used, given by the anatomical dimension of the pulp space itself. The increase between the 2 models is statistically significant (****P < 0.0001). (**E**) The coronal measurements show a consistent increase for the rat model with a mean width of 0.47 mm (±0.01 mm) (****P < 0.0001), which is almost twice the width of the bur itself. This increase is given by the prolonged incisors and posterior position of the rat molar, and hence, the bur is set at an angle, leading to an increased transversal pulp exposure. (**F**) The damage area measured at the maximum sagittal and coronal diameter is 9.7-fold bigger in the rat model compared to the mouse model (*P* < 0.0001). An unpaired 2-tailed *t* test was used to assess significant differences between groups, and error bars indicate SEM.

To evaluate the increase of the damaged area between the 2 species, µCT scans were assessed from mouse and rat teeth (Fig. 2A–C). Two-dimensional µCT images obtained at the maximum damage extent revealed 0.092 ± 0.004 mm (mouse) and 0.195 ± 0.01 mm (rats) at the sagittal plane (Fig. 2D) and 0.117 ± 0.008 mm (mouse) and 0.468 ± 0.005 mm at the coronal plane (Fig. 2E), resulting in an approximately 10-fold bigger damage area for rats (Fig. 2F).

### Range of Wnt-Activty in Tooth Pulp

**Figure 3.**
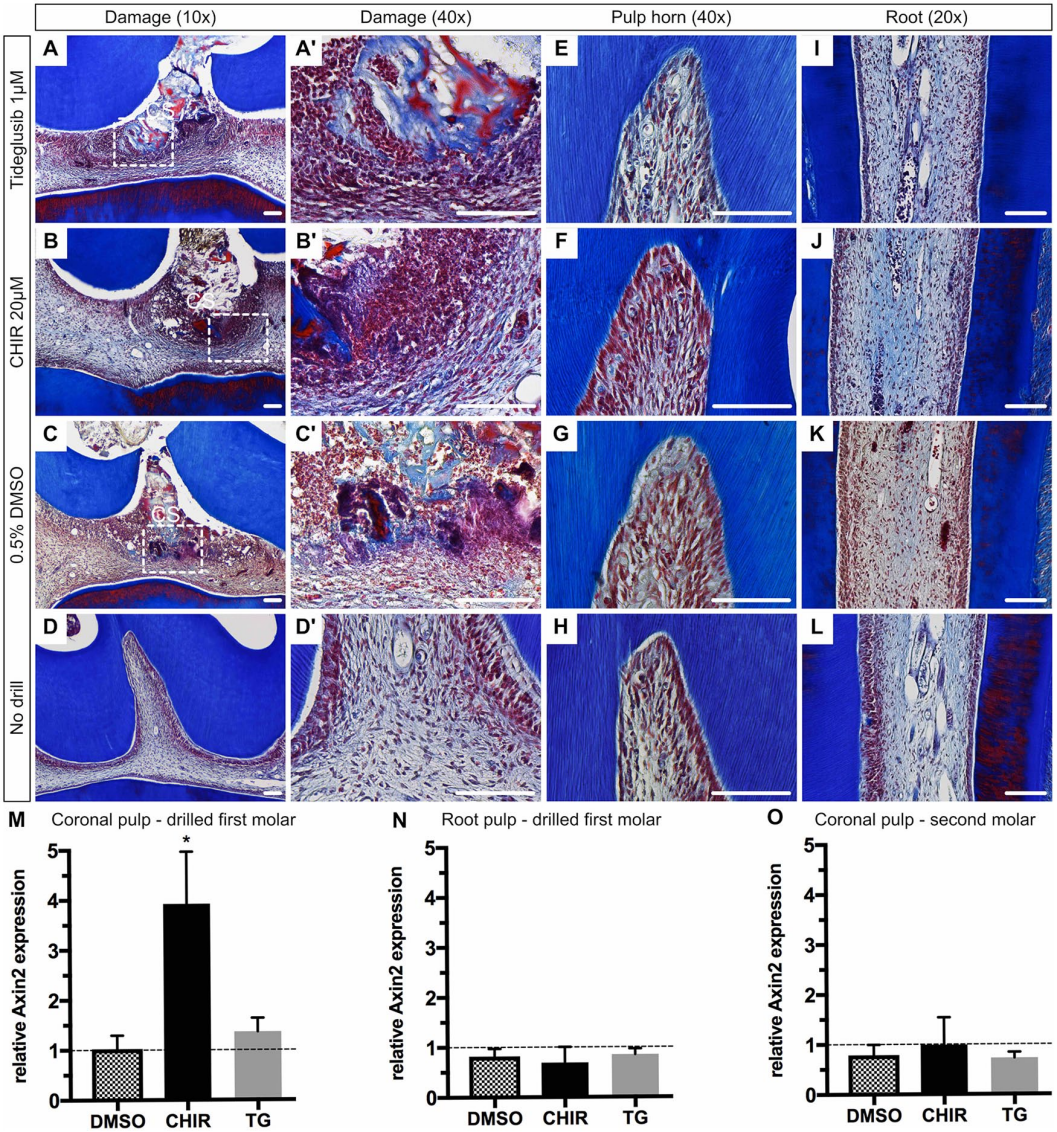
Range and activation of Wnt in tooth pulp. Masson’s trichrome staining of superior first molars 24 h postsurgery and of a nondrilled control. (**A–C**) The applied collagen sponge (CS), indicated by light blue staining (collagen), causes a localized immune reaction in the coronal pulp. The histology of the (**E–G**) mesial pulp horn and (**I–K**) mesial roots show no obvious difference compared to a (**H**, **L**) nondrilled control. (**M**, **N**) Relative *Axin2* expression of the coronal pulp and root pulp tissue of a drilled superior first molar 24 h after pulp exposure and drug placement compared to a no-drilled control (baseline *Axin2* expression, dotted line). (M) 20 µM CHIR99021 (CHIR) and 1 µM Tideglusib show increased messenger RNA expression of *Axin2* in the coronal pulp, but no elevated expression is present in the root pulp (N). (**O**) The coronal pulp tissue of the corresponding second molar (no damage) within the same treatment group shows similar gene expression to the nondrilled control (dotted line). A total of sixteen 7-wk-old adult Wistar rats (*n* = 4) were included with 3 animals for gene expression analysis (6 teeth) and 1 animal (2 teeth) for representative histology. An unpaired 2-tailed *t* test was used to assess significant differences between groups. Scale bars are equivalent to 100 µm and error bars indicate SEM (*P < 0.05).

Histological sections 24 h postdamage showed localized cell infiltration around the collagen sponge limited to the middle cusp (damage area) (Fig. 3A–C). Neither the remaining cusps nor the roots of drilled molars showed an alteration in histology (Fig. 3D–K).

To quantify the extent of Wnt-activty within the dental pulp, we extracted RNA from the coronal and root pulp tissues and assayed for relative gene expression analysis. Elevated *Axin2* expression was observed in the coronal pulp tissue of drilled molars using CHIR (3.9-fold; *P* *=* 0.0485) and TG (1.4-fold; *ns*) (Fig. 3M). Root pulp tissue of drilled teeth as well as coronal pulp tissue of neighboring teeth without damage (second molar) did not show any elevated *Axin2* expression (Fig. 3N, O).

### Composition of Wnt-Induced Reparative Dentine

**Figure 4.**
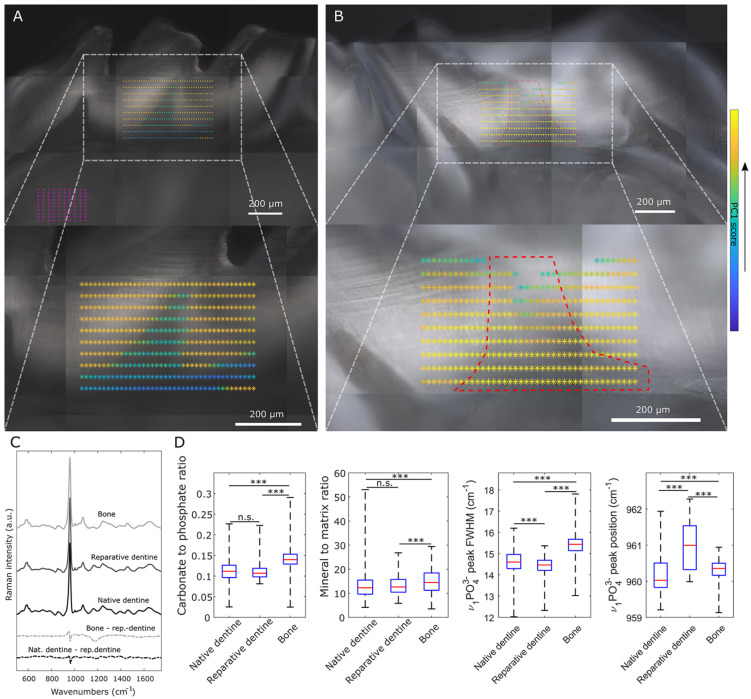
Comparison of reparative dentine with native dentine and bone by Raman microspectroscopy. (**A**) White light image of control tooth with first principal component (PC1) scores (false colors) of 400 Raman measurements. Principal component analysis was performed on all Raman spectra from 6 treated teeth and 1 control. High scores on PC1 show mineralized areas and low scores correlate with soft tissue. Magenta crosses indicate positions of Raman measurements of bone. (**B**) White light image of tooth with a defect that was treated with a drug-containing sponge showing high degree of mineralization across the defect site (red dashed line) as determined by PC1 scores. (**C**) Mean Raman spectra of bone (*n* = 700), reparative dentine (*n* = 199), and native dentine (*n* = 714). Difference spectra comparing bone and native dentine with reparative dentine are shown in the bottom of the panel. Spectra are offset on the *y*-axis for clarity. Spectra of reparative dentine were identified in tooth defects from white light images and high degree of mineralization (PC1) as shown in panel B. Spectra of native dentine were identified similarly from areas outside regions corresponding to pulp or defect. (**D**) Univariate analysis of Raman spectral features in native dentine (*n* = 714), reparative dentine (*n* = 199), and bone (*n* = 700). Carbonate to phosphate ratio determined from 1,070-cm^–1^ (ν_1_CO_3_^2–^) and 960-cm^–1^ (ν_1_PO_4_^3–^) peak areas and mineral to matrix ratio from (ν_1_PO_4_^3–^) and 853-cm^–1^ to 875-cm^–1^ (νC-C) peak areas. Kruskal-Wallis nonparametric test followed by Wilcoxon rank-sum test was used to detect statistical significance (***P < 0.001).

We applied Raman microspectroscopy in maps across normal and repaired mouse teeth to compare native dentine and bone with the reparative dentine formed. The PCA revealed that the first 5 principal components (PCs) accounted for 90% of the spectral variation. High scores for PC1 correlated with mineralized native dentine in controls (Fig. 4A) and were used as an indicator of both mineralization and the similarity of reparative to native dentine. The “heat maps” enable one to visually compare the chemical compositions of reparative and native dentine (Fig. 4B, Appendix Figs. 3 and 4). While mean difference spectra between bone and reparative dentine (Bone-rep.dentine) and native and reparative dentine (Nat.dentine-rep.dentine) contained an upward shift at ~960 cm^–1^ in both, the Bone-rep.dentine difference spectrum contained more features than the Nat.dentine-rep.dentine difference spectrum (particularly at ~1,200 cm^–1^), hypothesizing that reparative dentine is more akin to native dentine than bone (Fig. 4C). To confirm this, we carried out a series of univariate analyses. The proline 853-cm^–1^ to 875-cm^–1^ (νC-C) band (specific to collagen) and the 1,070-cm^–1^ (ν_1_CO_3_^2^) band (sensitive to B-type carbonate substitution in the apatite) were used to measure matrix and carbonate mineral to matrix and carbonate to phosphate ratios (Mandair and Morris 2015). The ratios were not significantly different between native and reparative dentine, but they were both significantly different from bone. To better understand the significance of the upward shift at 960 cm^–1^, we carried out analyses of mineral crystallinity. In mineralized tissue, an upward shift in the position of the ν_1_PO_4_^3–^ peak is associated with a more crystalline apatite (Tarnowski et al. 2002). We found the position of the ν_1_PO_4_^3–^ peak in reparative dentine was significantly higher than in native dentine and bone (Fig. 4D; Appendix Fig. 3). This change in crystallinity was confirmed by measuring the FWHM of the ν_1_PO_4_^3–^ peak, which is inversely proportional to the mineral crystallite c-axis length (Mandair and Morris 2015), and was lower in reparative dentine compared to both bone and native dentine.

## Discussion

The treatment of dental caries that results in pulp exposure is currently managed by replacing lost dentine with inorganic calcium-containing materials such as calcium hydroxide and MTA that remain in the crown. Although there is some natural regeneration of dentine (reparative dentine), this is minimal and only forms a thin layer below the MTA (Bakhtiar et al. 2017). Since this dentine is formed directly from new odontoblast-like cells that differentiate from resident stem cells in the pulp (Babb et al. 2017), it is conceivable that overstimulation of stem cell activity might result in increased odontoblast differentiation resulting in more robust regenerative dentine formation.

In humans, pulp exposure can occur during caries removal of deep carious lesions, during prosthetic rehabilitation (tooth preparation), or following trauma. To treat such vital cases, a partial pulp removal of 1-mm to 2-mm depth has been clinically established (Cvek 1978; Chailertvanitkul et al. 2014; Ricucci et al. 2019). The lesion size of a partially removed pulp in humans will be given by the clinical situation, but a recent study found the majority of exposed cases during caries removal exhibited a surface damage area between 0.01 mm^2^ and 2 mm^2^ (Chailertvanitkul et al. 2014). Using these dimensions, the potential cubic volume (πr^2^h) needed to be repaired in humans was estimated as 0.012 mm^3^ with *r* = 0.0625 mm (smallest bur available Ø 0.125 mm) and h = 1 mm to 4.02 mm^3^ (*r* = 0.8 mm; h = 2 mm). In rats, we were able to completely repair a surface damage area of 0.1 mm^2^ and volume of 0.055 mm^3^, which mimics a small exposed pulp area in a clinical situation. Compared to the previous published data in mice (Neves et al. 2017), we restored a 10-fold bigger exposed pulp area. Although the extent of damage in rats does not represent the size of large lesions in humans, the successful scaleup of reparative dentine formation in vivo highlights a promising potential for translational research approaches.

While highly mineralized dentine like peritubular dentine reveals a Young’s modulus of 40 to 42 GPa, weakly mineralized dentine like intertubular dentine exhibits only 17 GP (Ziskind et al. 2011; Zhang et al. 2014). Crack formation is observed more frequently in coronal dentine along the peritubular dentine, whereas root dentine shows a higher fracture resistance due to the lack of peritubular dentine (Wang 2005; Zhang et al. 2014). The chemical mineral composition analysis by Raman microspectroscopy revealed that reparative dentine was similar to native dentine, both being different from surrounding alveolar bone. The only difference between regenerated dentine and native dentine was slightly higher apatite crystallinity, which may be associated with an imbalance between the formation of more intertubular versus peritubular-like dentine.

To prevent systemic side effects of the highly potent small molecules acting as Wnt agonists, a local application and therapeutic effect are mandatory if used for reparative dentinogenesis. In rats, we applied a collagen sponge enriched with 0.1 µL of 1 µM TG (0.03 ng) or 20 µM CHIR (0.93 ng) in direct contact to the damaged pulp tissue, subsequently triggering *Axin2* gene expression. We showed that the activity range of CHIR and TG was restricted to the coronal pulp. Both the adjacent root pulp tissues and the pulp tissue of a neighboring tooth showed no elevated *Axin2.* Corresponding histology revealed cellular changes in close proximity to the collagen sponge with no obvious differences in the mesial or distal pulp horn or the mesial root pulp tissue. The average blood volume of the rats used for this experiment is about 12 mL (mean weight 191.3 ± 11.5 g) according to the NC3R estimation (64 mL blood/kg body weight) (National Center for the Replacement Refinement and Reduction of Animals in Research 2019). Hence, if the drugs enter the systemic circulation completely, concentrations would reveal 0.08 pmol/L for TG and 1.6 pmol/L for CHIR. These concentrations are well below the clinically administered dosages to patients enrolled in clinical trials even if increased to account for human size lesions as mentioned above (del Ser et al. 2013; Lovestone et al. 2015).

The ultimate goal of regenerative medicine is to restore the original properties, functions, and compositions of lost tissues. In terms of GSK-3 inhibitor-triggered reparative dentinogenesis, we are still not able to truly “regenerate” the lost tissue, but we are able to deliver a reproducible technique that allows us to form a reparative dentine close to native dentine compositions at defect sizes translatable to small human lesions.

## Author Contributions

L.K. Zaugg, contributed to conception, design, data acquisition, and analysis, drafted and critically revised the manuscript; A. Banu, D. Chandrasekaran, C. Salzlechner, contributed to design and data acquisition, drafted and critically revised the manuscript; A.R. Walther, contributed to design, data acquisition, and analysis, drafted and critically revised the manuscript; R.C. Babb, contributed to conception, design, and data acquisition, drafted and critically revised the manuscript; M.A.B. Hedegaard, contributed to design, data acquisition, analysis, and interpretation, drafted and critically revised the manuscript; E. Gentleman, P.T. Sharpe, contributed to conception, design, data analysis, and interpretation, drafted and critically revised the manuscript. All authors gave final approval and agree to be accountable for all aspects of the work.

## Supplemental Material

DS_10.1177_0022034520908593 – Supplemental material for Translation Approach for Dentine Regeneration Using GSK-3 Antagonists Supplemental material, DS_10.1177_0022034520908593 for Translation Approach for Dentine Regeneration Using GSK-3 Antagonists by L.K. Zaugg, A. Banu, A.R. Walther, D. Chandrasekaran, R.C. Babb, C. Salzlechner, M.A.B. Hedegaard, E. Gentleman and P.T. Sharpe in Journal of Dental Research
