# Supplementary material for: Translation Approach for Dentine Regeneration Using GSK-3 Antagonists
Source: J Dent Res. 2020 Mar 10;99(5):544–51. doi: 10.1177/0022034520908593 (PMC7534023; doi:10.1177/0022034520908593)
Supplement: DS_10.1177_0022034520908593 – Supplemental material for Translation Approach for Dentine Regeneration Using GSK-3 Antagonists [file DS_10.1177_0022034520908593.pdf]

Translation Approach for Dentine Regeneration Using GSK-3 Antagonists

L.K. Zaugg, A. Banu, A.R. Walther, D. Chandrasekaran, R.C. Babb, C. Salzlechner, M.A.B. Hedegaard, E. Gentleman, and P.T. Sharpe

Appendix

Supplementary materials

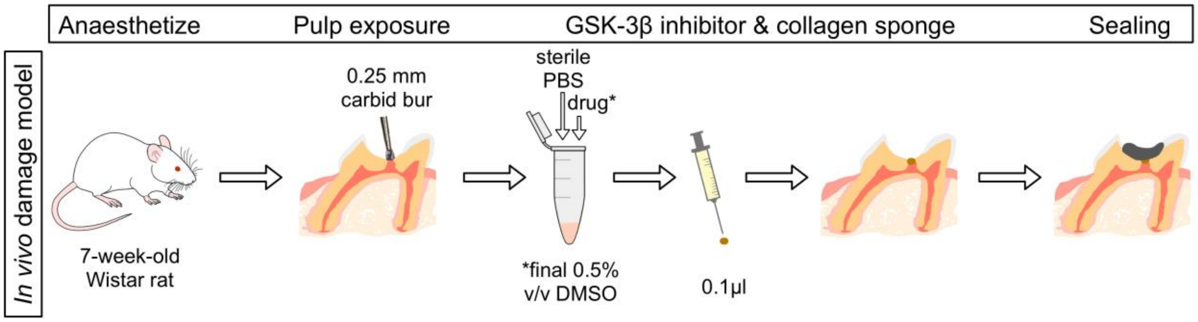

Appendix Fig. 1. *In vivo* model to study reparative dentinogenesis. Schematic diagram of *in vivo* pulp damage model of 7-week-old male Wistar rats; a carbide bur (Ø 0.25mm) mounted on a high-speed hand-piece was used to expose the pulp of the middle cusp of anaesthetized rats. All drugs were dissolved in DMSO and diluted to a final concentration of 0.5% w/v DMSO in PBS according to the selected concentration. Bleeding control was obtained with sterile paper points and a 1mm^3 collagen sponge enriched with 0.1µl drug was placed in direct contact to the pulp tissue. The tooth was sealed with glass ionomer cement subsequently. Short (24h) and long (4 weeks) time points were selected for gene expression and histology/µCT analysis, respectively.

Primary rat cell culture from pulp explants

Primary pulp cell cultures from explants of maxillary and mandibular first molars of 5-day-old, Wistar rats were established for preliminary drug testing assays (cell viability assessment, dose and time dependent relative *Axin2* gene expression).

A standard medium (α MEM, UltraGlutamine, Lonza) enriched with 15% FBS (Gibco) and 1% Antibiotic / Antimycotic Solution (ABAM, Sigma) was prepared. Ten 5-day-old Wistar rats were sacrificed by cervical dislocation and the non-erupted first molars were extracted immediately under aseptic conditions using 20G needles and microscopes. Teeth were washed twice in ice cold PBS (Sigma) and the pulp tissue was removed from the developing teeth using fine straight tip tweezers and 27G needles. The pulp explants were placed in a 24-well culture dish (one pulp per well) and kept on ice. After initial attachment the explants were covered with 400µl medium and incubated (37°C, 5% CO_2/95% O_2, 100% humidity). Medium was replaced on day 2 (500µl), thereafter 2-3 times a week. Cells were passaged at 80% confluence with trypsin (TrypLE Express, Gibco) and expanded (Appendix Fig. 2A).

Cell viability assessment

Primary pulp cells cultured from 5 day-old rats were plated in 96 well plates at 10’000 cells/well and incubated (37°C, 5% CO_2/95% O_2, 100% humidity) using standard culture medium. After 24h of incubation, the medium was replaced with 100µl conditioned (drug + media) or control medium (medium alone) for another 24h. The following concentrations of CHIR and TG were tested: 1mM, 100µM, 50µM, 20µM, 10µM, 1µM, 100nM and 10nM. Stock concentrations were diluted in DMSO in order to equalize the final amount of DMSO per well. Concentrations ranging from 10nM to 20µM comprised 0.5µl DMSO + 99.5µl medium (0.5% v/v), 50µM and

100µM comprised 2.5µl DMSO + 97.5µl medium (2.5% v/v) and 1mM comprised 25µl DMSO + 75µl medium (25% v/v). DMSO only was tested at the above-mentioned concentrations. A control plate was set up with the same conditioned / control medium without cells in order to account for chemical interference of test compounds with the MTS assay (CellTiter 96 AQuous, Promega). After the 24-hour incubation period, representative images (Appendix Fig. 2, B-J) were taken and 20µl of MTS tetrazolium compound were added to each well using multichannel pipettes. Plates were incubated for another 2.5h and the absorbance of the produced formazan was measured at 490nm using a 96-well plate reader (ClarioStar, BMG Labtech). Background absorbance obtained from control plates was subtracted individually and data was normalized. The experiment was performed in duplicates and repeated independently.

Time and dose dependent GSK-3 inhibitor testing in-vitro

Primary pulp cells were plated at a density of $0.1x10^6$ cells/ml in 24-well culture dishes and incubated for 24h (37°C, 5% CO_2/95% O_2, 100% humidity). Passage numbers for all experiments were between P6 and P9. Cells were treated with 10µM and 20µM CHIR, 1µM and 10µM TG or DMSO diluted to a final concentration of 5µl drug/DMSO per 1ml medium (0.5%v/v). After 0.5h, 1h, 4h, 6h and 24h incubation, wells were washed 3x with ice cold PBS. For RNA extraction, cells (3 wells per group) were harvested using 350µL RLT buffer enriched with 1% β-mercaptoethanol (Rneasy Mini Kit, Quiagen) and stored at -80°C. RNA was extracted using the Rneasy Mini Kit (Quiagen) according to the manufacturer's instructions. RNA quantification was measured by NanoDrop spectrophotometer (Thermo Fisher) and the absorbance ratios of 260/280 and 260/230 were recorded for quality control. Revers transcription and real time qPCR analysis was performed with *Axin2* as readout. All experiments were repeated independently in triplicates.

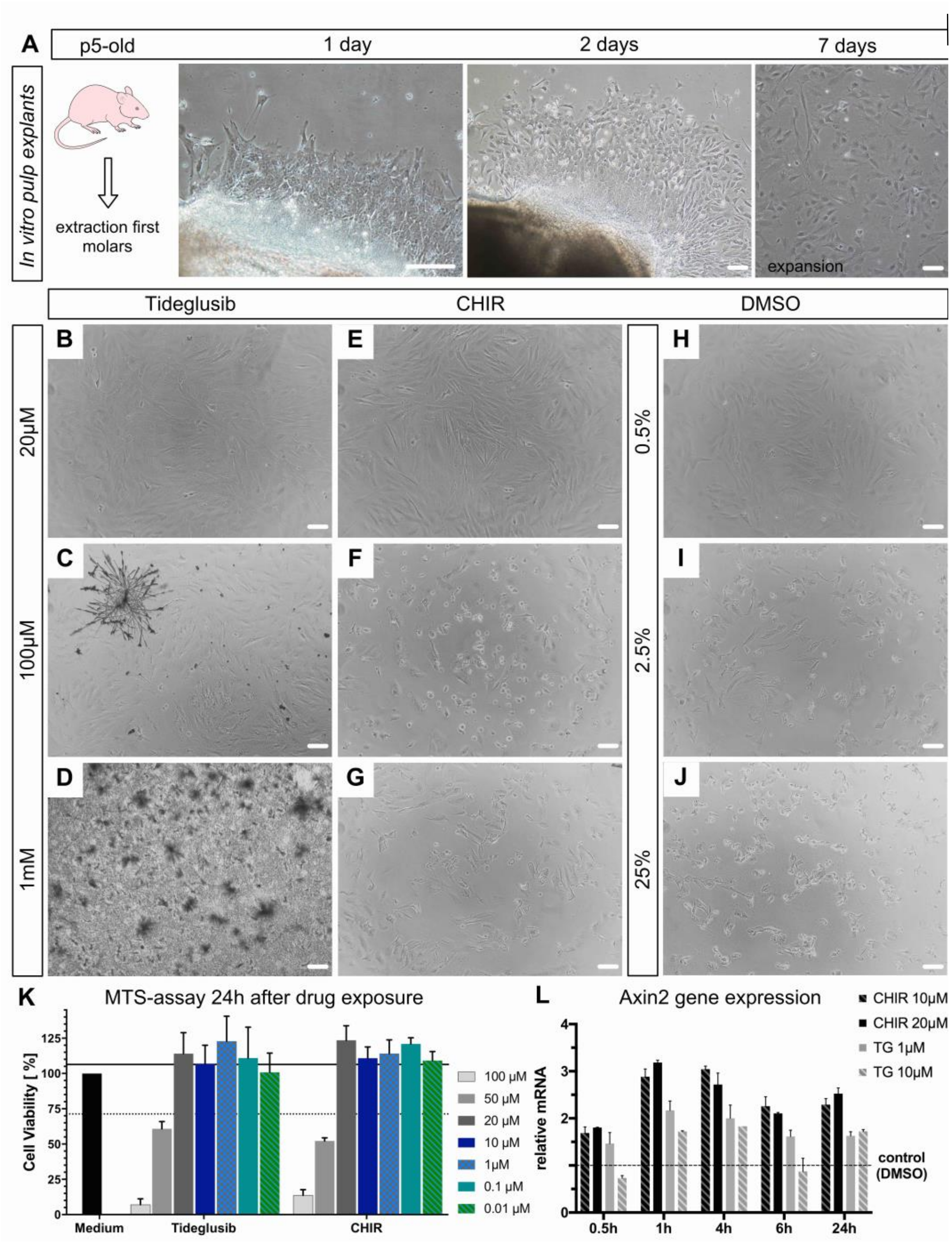

Appendix Fig. 2. *In vitro* model to study Wnt/β-catenin signaling in primary rat pulp cell explant cultures. (A) Primary pulp cell cultures from explants of maxillary and mandibular first molars of 5-day-old Wistar rats to investigate cell viability and to investigate dose and time

dependent Wnt/β-catenin signaling *in vitro*. Each pulp explant was placed in a separate well and monitored for cell outgrowth. Cells were passaged after 5 days and reached 80% confluence after 7 days for further expansion. **(B-J)** Cell viability assessment of primary rat pulp cell culture of 5-day-old rats 24h after treating the cells with Tideglusib **(B-D)**, CHIR **(E-G)** and three concentrations of DMSO **(H-J)**; all cells remain viable at 20µM resp. 0.5% DMSO, whereas concentrations of 50µM and 100µM (equivalent 2.5% DMSO) show decreased cell viability. **K)** MTS viability assay indicates increased viability at concentrations of 1µM Tideglusib and 20µM CHIR; black horizontal line represents 0.5% DMSO, dotted line 2.5% DMSO respectively. **(L)** Relative *Axin2* gene expression normalized against DMSO-control (dotted line); Wnt activity reaches its peak after 1h and stays elevated up to 24h. Scale bars indicate 100µm.

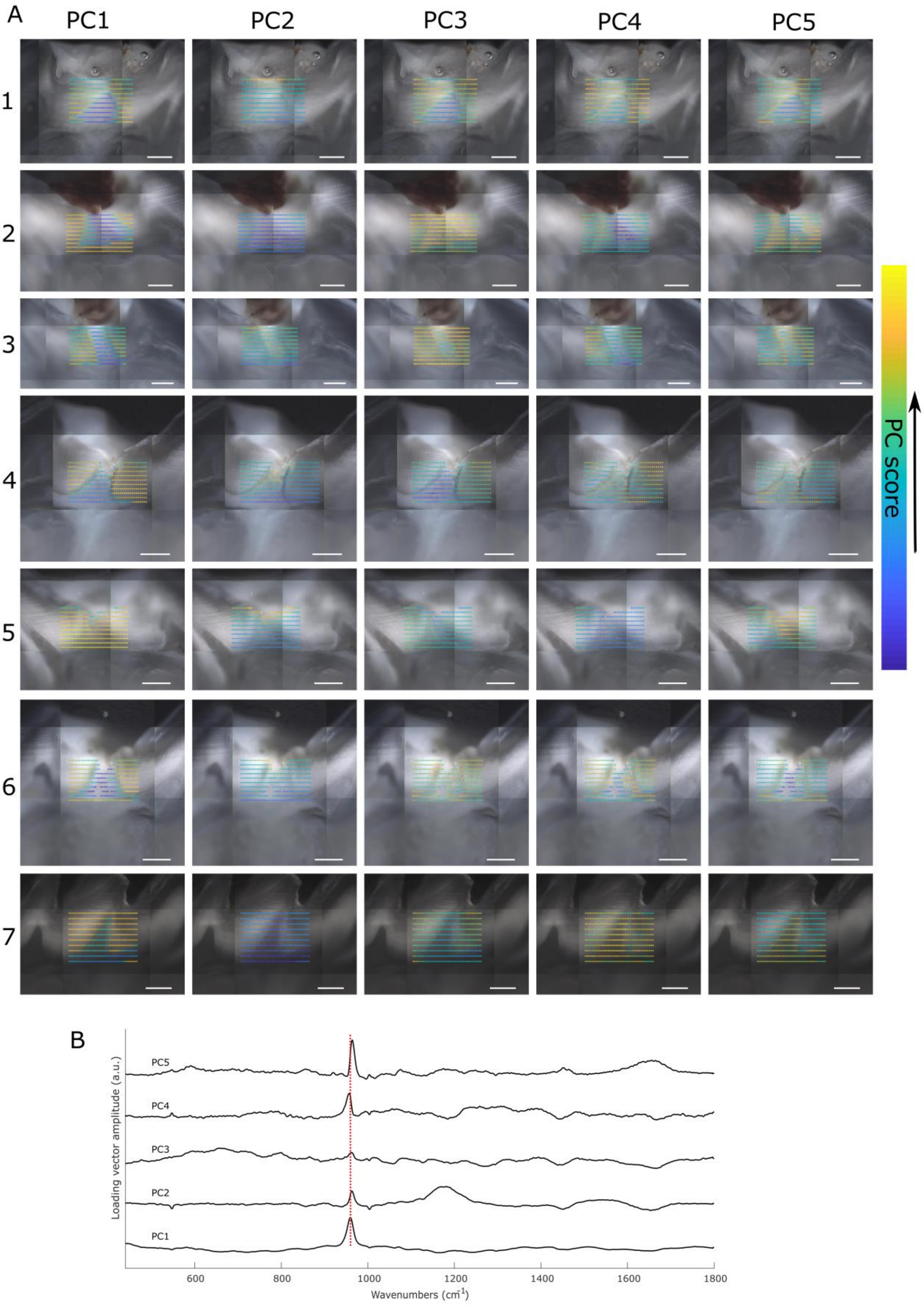
A
PC1
PC2
PC3
PC4
PC5
1
2
3
4
5
6
7
PC score
B
PC5
PC4
PC3
PC2
PC1
Loading vector amplitude (a.u.)
600
800
1000
1200
1400
1600
1800
Wavenumbers (cm-1)

Appendix Fig. 3. Principal component analysis (PCA) of Raman microspectroscopic measurements. (A) White light images of treated (rows 1-6) and control (row 7) teeth overlaid with false color heat maps showing scores of Raman spectral observations for the first five principal components (PC 1-5, columns). PC1-5 accounted for more than 90% of the spectral variation in the dataset. Scale bar = 200 µm. **(B)** Loading vectors of first five principal components, shifted vertically for clarity. The red dashed line indicates the position of the $\nu_1PO_4^{3-}$ peak at ~960 cm^{-1}. The principal component analysis decomposes the Raman spectral observations into linear combinations of loading vectors, weighted by the scores. Therefore, as Raman spectra with high PC1 scores are dominated by the 960 cm^{-1} $\nu_1PO_4^{3-}$ apatite peak (indicated by the PC1 loading vector), and correlate with the native mineralized dentine in the control sample (row 7), PC1 can be considered as an indicator of mineralization. In this context, we observed reparative mineralized dentine across the entire defect in the tooth in row 5, but in the tooth in row 1, mineralized dentine only partly sealed the defect. PC2 correlates primarily with the glass ionomer cement capping, while high scores of PC3 are apparent in teeth, which did not retain their capping (row 2 and 3). PC4 and 5 loading vectors indicate slightly different apatite species, as indicated by either downwards or upwards shifts of the 960 cm^{-1} peak, respectively. In PC5, in particular, the shift in the position of the 960 cm^{-1} peak in areas of repaired dentine support our finding from the univariate analysis that the newly formed dentine is slightly more crystalline than native dentine.

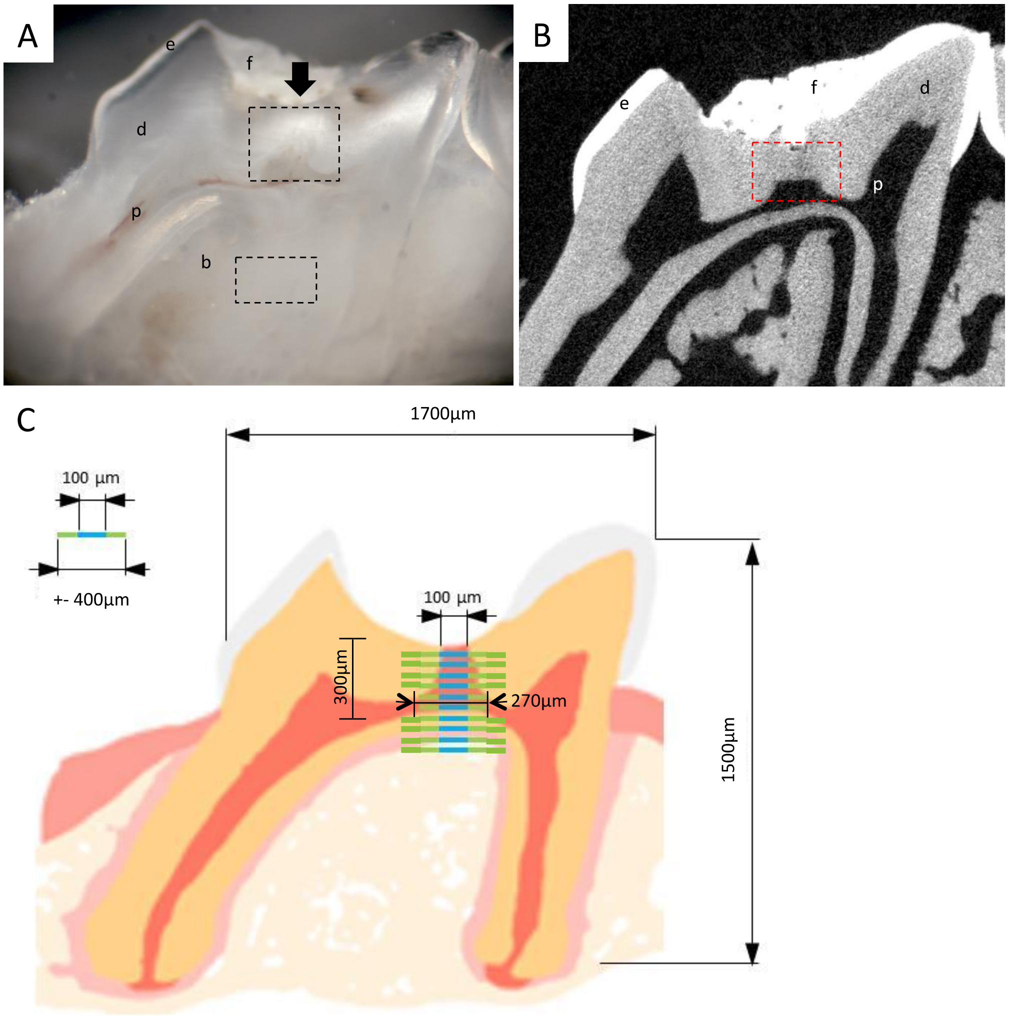

Appendix Fig. 4. Sample orientation and dimensions for Raman microspectroscopy analyses. (A) Bright field image of a mouse upper first molar 6 weeks after the tooth was damaged and a collagen sponge enriched with 50nM BIO was placed in the defect. The upper square indicates the area that was analyzed by Raman microspectroscopy, which was chosen, as it comprised native dentine, newly formed dentine, and pulp tissue. The lower square indicates the area analyzed to compare the reparative/native dentine to surrounding alveolar bone. The arrow indicates the position at which the tooth was damaged. **(B)** Corresponding µCT image of the plane of the tooth shown in (A), confirming the formation of a dentine bridge (red square). The µCT image allowed us to confirm the dimensions of the damaged and repaired areas in order

to define the area for Raman microspectroscopy measurements. **(C)** Schematic of the tooth and the dimensions that were analyzed using Raman microspectroscopy. The upper first mouse molar is around 1700µm wide and 1500µm high at the central sagittal section. In our damage model, the width of the site of pulp exposure is ~100µm, while the basis of the pulp horn measures around 270µm. The height of this area is ~300µm. In order to collect Raman spectra of both native dentine and reparative dentine, a minimal line length of 400µm was chosen. An extra 50µm on each side was added to assure we collected a sufficient number of native dentine spectra, creating a final area of ± 500 x 300 μm^2 with 400 spectra being collected for each tooth. f = glass ionomer filling, e = enamel, d = dentine, p = pulp, b = bone.
